# Complement-targeting therapeutics for ischemia-reperfusion injury in transplantation and the potential for *ex vivo* delivery

**DOI:** 10.3389/fimmu.2022.1000172

**Published:** 2022-10-19

**Authors:** Isabel F. Delaura, Qimeng Gao, Imran J. Anwar, Nader Abraham, Riley Kahan, Matthew G. Hartwig, Andrew S. Barbas

**Affiliations:** ^1^ Department of Surgery, Duke University School of Medicine, Durham, NC, United States; ^2^ Division of Cardiovascular and Thoracic Surgery, Duke University Medical Center, Durham, NC, United States

**Keywords:** complement inhibitor, ischemia-reperfusion injury, *ex vivo* delivery, organ transplantation, classic pathway

## Abstract

Organ shortages and an expanding waitlist have led to increased utilization of marginal organs. All donor organs are subject to varying degrees of IRI during the transplant process. Extended criteria organs, including those from older donors and organs donated after circulatory death are especially vulnerable to ischemia-reperfusion injury (IRI). Involvement of the complement cascade in mediating IRI has been studied extensively. Complement plays a vital role in the propagation of IRI and subsequent recruitment of the adaptive immune elements. Complement inhibition at various points of the pathway has been shown to mitigate IRI and minimize future immune-mediated injury in preclinical models. The recent introduction of *ex vivo* machine perfusion platforms provides an ideal window for therapeutic interventions. Here we review the role of complement in IRI by organ system and highlight potential therapeutic targets for intervention during *ex vivo* machine preservation of donor organs.

## 1 Introduction

Transplantation provides a curative treatment for end-organ pathologies; however, its utility is limited by critical organ shortages. Over the last decade, organ waitlists have continued to grow faster than transplants performed, with 161,758 patients awaiting transplant in the United States in 2020 (1). Accordingly, strategies to combat the increasing organ shortage have focused on expanding the donor pool to include organs from extended criteria donors, the definition of which varies by organ type. In kidney transplantation, extended criteria donors includes donors with acute kidney injury (AKI) (2, 3) and those with a high Kidney Donor Profile Index (KDPI) (4, 5). KDPI considers donor demographics (height, weight, age, ethnicity), comorbidities (diabetes, hypertension), laboratory parameters (creatinine, hepatitis C serology), and other characteristics such as cause of death and donation after circulatory death (DCD) status. Marginal livers—those donor organs with characteristics associated with early graft failure and worse overall outcomes—include allografts with significant steatosis, DCD, and those from elderly and Hepatitis C positive donors (6, 7). Extended criteria heart donors are those older than 65, or with significant comorbidities such as poorly controlled diabetes, renal insufficiency, and peripheral arterial disease (8). In lung transplantation, extended criteria donor fulfills two or more of the following criteria: age ≥ 55, smoking history ≥ 20 pack-year, pO2 ≤ 300, diabetes, purulence found on bronchoscopy, bloodstream infection, and abnormal chest x-ray (9). Among extended criteria organs, transplant using DCD organs has substantially broadened the donor pool. DCD currently accounts for 25% of kidney deceased organ donation (1), 10% of liver donation (10) and 7.4% of all lung donations (11). Since the first DCD heart transplant in the US in 2019, 3.3% of all heart transplants in 2020 used DCD organs (12).

However, increased utilization comes at a cost of potentially increased incidence of early graft dysfunction and compromised long-term outcomes. Both DCD (13) and extended criteria donor (14) kidney transplants result in a higher incidence of graft loss and delayed graft function (DGF). DGF is further associated with increased graft loss and acute rejection in kidney transplant (15). In both liver (16, 17) and heart transplants (8), marginal donors are associated with higher rates of graft loss and recipient mortality. While inclusion of marginal organs in transplantation may provide benefit over remaining on the waitlist, it is associated with compromised short- and long-term outcomes compared to standard criteria donor organs.

Marginal organs are more vulnerable to insults such as ischemia-reperfusion injury (IRI); thus, particular attention is paid to minimizing these insults in DCD and extended criteria organ transplantation. One approach is to minimize cold ischemic time (CIT), which is typically shorter in DCD compared to DBD transplants (18). Recent improvements in liver transplantation outcomes using elderly donors organs have been associated with reduction in cold ischemia time as well (19). Another strategy to minimize IRI is the delivery of therapeutic agents targeting various pathways implicated in IRI pathogenesis, such as the complement system, a multi-functional component of the innate immune system. Various complement-targeting therapeutics have recently become clinically available, offering an opportunity for application to organ transplantation (20). Concurrently, *ex vivo* machine perfusion technology has been developed for organ preservation and evaluation. This platform may be utilized for the delivery of various therapeutics. In this review, we first outline the role of complement in IRI, and highlight the current evidence evaluating complement inhibition as preventative therapy for IRI. We also review the advantages of and available literature on *ex vivo* delivery of complement inhibition during donor organ preservation. The review focuses predominantly on kidney transplant, given that most studies have been performed in kidney transplant; however, we will highlight studies performed in other organs as well as important differences in complement inhibition across organ systems.

## 2 The role of complement system in IRI

### 2.1 Overview of the complement system

**Figure 1:**
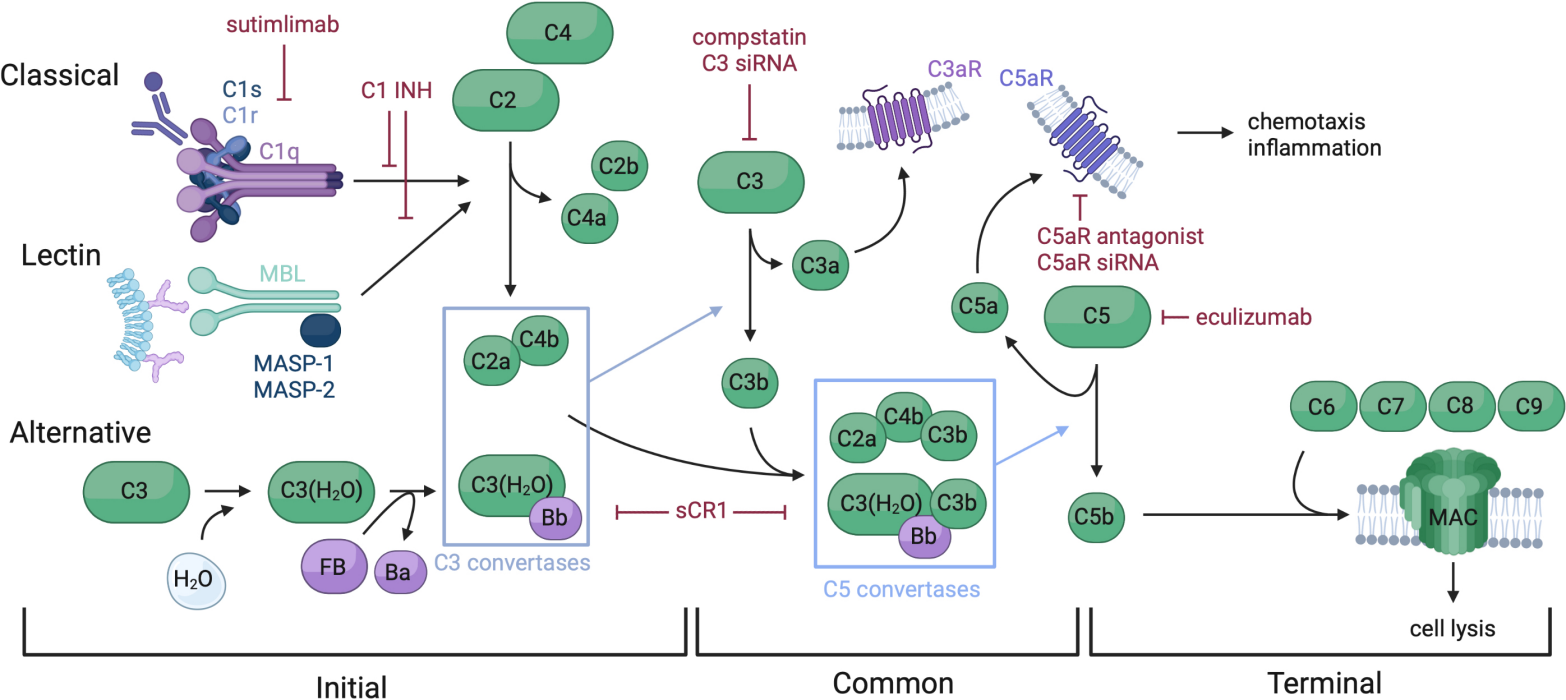
*Overview of the complement system and therapeutic targets.* The complement system is comprised of soluble, membrane bound, and regulatory proteins, and may be activated via three distinct pathways: classical, lectin, and alternative. These pathways converge on the common pathway with the formation of a C3 convertase which splits C3 into C3a and C3b. C3b complexes to the C3 convertases to form a C5 convertase, which splits C5 and triggers assembly of the MAC, making up the terminal pathway. Complement functions include cell lysis via MAC insertion into cell membranes and chemotaxis and inflammation regulated by split proteins C3a and C5a. Therapeutics targeting several complement components have been developed, including C1 INH which inactivates C1s, C1r and MASP-1 and -2, thus preventing spontaneous activation of the classical and lectin pathways. Sutimlimab is an anti-C1s mAb approved for cold agglutinin disease. sCR1, and versions of this molecule such as mirococept inhibit C3 and C5 convertases, among other functions. Multiple C3 targeting therapeutics have been developed, such as compstatin and C3 siRNA. C5 and C5aR have been targeted by drugs such as eculizumab (anti-C5 mAb), C5aR antagonist, and C5aR siRNA.

Complement is a key driver of IRI pathogenesis (21, 22). In brief, the complement system may be activated through three initial pathways: classical *via* binding of C1q to the Fc region of antibody, lectin *via* recognition of mannose-binding lectins (MBL) and activation of MASP proteases, or alternative *via* C3 hydrolysis (**Figure 1**). All three initial pathways converge on the formation of a C3 convertase and the common pathway. Anaphylatoxins and split products C3a and C5a serve several purposes, including inflammatory mediation and neutrophil chemotaxis. Split product C5b begins the terminal pathway by complexing with proteins C6-9, forming the membrane attack complex (MAC), which facilitates cell lysis. Under normal homeostasis, the complement system is strictly regulated to prevent unwanted tissue damage. Soluble proteins, such as C1 esterase inhibitor (C1 INH), Factor I and H, Clusterin and vitronectin, and membrane-bound proteins, such as membrane cofactor protein (CD46), Decay-accelerating factor (CD55), CD59 and complement receptor I, regulate various steps of the three complement pathways, inhibiting further propagation of the cascade when activated (23–25).

### 2.2 Complement activation in deceased donors

From an organ transplantation perspective, the disturbance of such a delicate balance occurs immediately following brain death in organ donors. Systemically, increased levels of C5a (26–28) and C5b-9 were found in the serum of deceased donors, compared to living donors. Upon reperfusion, there appears to be a transient release of C5b-9 detected in recipient serum (29). At the organ level, most complement proteins are synthesized in the liver, while kidney and lung are two of the few organs capable of synthesizing complement locally. Exploring the differences in gene expression profiles between living and deceased donors, Naesens et al. found significant over-expression of many complement components, including C1q, C1s, C1r, C2, C4 and Factor B, a finding later confirmed using microarray technique by Damman et al. (30). In a syngeneic rat kidney transplant model, local C3 deposition on endothelium and glomeruli was found one hour post reperfusion and persisted until posttransplant day 5 in DBD transplant but not living donor kidneys (31). Additionally, higher renal C3 gene expression was observed in DBD rats compared to living donors (32).

The etiology of complement activation in deceased donors is thought to be related to the release of endogenous damage-associated molecular pattern molecules (DAMPs) and sterile inflammation (33). Although most studies have examined complement activation in standard DBD donors, intuitively, the degree of inflammation and thus complement activation may be more profound in DCD and extended criteria donors. Accordingly, comparisons of transcriptional profiles and histology have shown increased activation of inflammatory chemokines, complement, and coagulation in DCD and extended criteria kidneys, compared to standard criteria donors (34, 35).

### 2.3 Complement activation during ischemia and reperfusion

IRI is inevitable to a degree in the process of organ transplantation, which may be further exacerbated depending on procurement method and storage. The underlying pathways driving IRI are numerous and affect many cellular components: initial ischemia leads to hypoxia, ATP depletion, and subsequent tissue necrosis. Injury is worsened during reperfusion through the production of ROS (36, 37). IRI injury results in the release of DAMPs, which in turn activate complement, perpetuating an ongoing inflammatory cycle. The particulars of complement modulation and pathogenesis of IRI vary widely depending on organ system (21, 38–40).

It is well established that complement mediates renal IRI. Mice lacking common pathway components such as C3 or C5, or C6 knockout mice unable to assemble membrane attack complex are protected from IRI (41). While the involvement of the classical pathway in IRI pathogenesis is well described (42), recent studies indicated an essential role for both lectin and alternative pathways. In murine models, mice deficient in Factor B (alternative pathway) (43), collectin-11 or Mannan-binding lectin (lectin pathway) (44, 45) were protected from renal IRI. Conversely, RAG-1 knockout animals deficient in Ig remained susceptible to IRI (46). Lastly, although the liver synthesizes the majority of circulating complement, complement proteins locally synthesized in the kidney plays an important role in injury. In a mouse kidney transplant model, Pratt et al. showed that donor allografts unable to synthesize C3 experienced reduced allograft rejection when transplanted into recipients with intact complement production; however, transplant of a wild-type kidney into C3 knockout mice did not see the same benefit (47). In human studies, donor C3 allotype was found to influence clinical kidney transplant outcomes (48).

In cardiac IRI models, injury is correlated with C3d deposition, and mouse studies have shown decreased tissue injury, troponin I levels, and immune cell infiltration with C3 knockout (49). In contrast to renal IRI, the classical pathway appears to be the prominent driver of myocardial IRI, with higher rates of IgM and C4d deposition in DBD hearts (49, 50).

The liver is unique as it is the primary location of complement synthesis and contains the largest collection of phagocytic cells in body. Liver tissue also expresses multiple complement receptors such as CR1, CR3, CR4, and C5aR, as well as CR immunoglobulin (51). Complement activation plays a key role in mediating hepatic IRI, as C6-knockout mice produce lower levels of pro-inflammatory cytokines, resulting in improved liver function and less IRI (52). Interestingly, complement activation products C3a and C5a are also key in liver regeneration. In a mouse 70% partial hepatectomy model, C3- and C5-deficient mice succumb to liver failure, a phenotype rescued by the reconstitution of effector molecules C3a and C5a (53, 54). As a result, the use of complement inhibition for prevention and treatment of hepatic IRI must be carried out thoughtfully so as not to compromise liver regeneration.

Complement activation in lung IRI has been recognized and linked to primary graft dysfunction (PGD). Various complement inhibitors have been tested in this setting in both animal models and human studies (55). Shah et al. observed an increase in plasma C5a and C4a in patients with PGD (56) and Westall et al. described significant C3d and C4d staining associated with PGD (57). It is likely all three complement pathways are involved in pulmonary IRI, as increased complement products from all three pathways were found in BAL from patients with PGD compared to those without (58). Like the kidney, the lung is capable of synthesizing complement locally: type II epithelial cells synthesize and secrete complement proteins C2, C3, C4, C5 and factor B (59).

Overall, complement pathways behave differentially across different organ systems in the pathogenesis of IRI, posing unique challenges for complement blockade-based therapeutics.

## 3 Systemic complement blockade for IRI

### 3.1 Pre-clinical models

#### 3.1.1 Kidney

The complexity of the complement system offers many therapeutic targets, which fall into three broad categories: complement cascade proteins, complement receptors, and complement regulatory proteins that naturally counter complement activation. Many have targeted the common pathway of the complement cascade. In two earlier studies, Zheng et al. used small interfering RNA (siRNA) to silence complement proteins—namely C3 and C5aR—in a murine renal IRI model. Their initial study demonstrated successful C3 and caspase 3 silencing when siRNA was delivered 48 hours prior to the induction of ischemia. Upregulation of C3 was seen only in controls, while the treatment group had lower BUN and creatinine and prolonged mouse survival. Histological exam showed decreased tubular infarction, immune cell infiltration, and necrosis in mice treated with a combination of C3 and caspase 3 siRNA, as well as an 87% reduction in total injury area (60). They went on to test C5aR inhibition using the same RNA interference approach, demonstrating less injury with C5aR inhibition compared to controls (61). Another approach to C5aR antagonism—the use of an acetate salt compound—resulted in improved clinical renal function in a rat model of renal IRI. TNF-alpha was decreased in treated animals, and biopsies showed decreased severity of tubular injury. In comparison to C5 blockade, C5aR antagonism did not result in inhibition of MAC assembly, as demonstrated by comparable hemolytic assay results in the treatment and control groups (62).

Additionally, studies have utilized complement receptor type 1 (CR1), also known as Cb/C4b receptor, which accelerates the degradation of C3 and C5 convertases, as well as activates factor I, leading to degradation of C3b and C4b (63). In a murine renal IRI model, Hameed et al. demonstrated improved serum creatinine, reduced neutrophil influx and superoxide production in the sCR1 treatment group (64). Bongoni et al. evaluated CSL040, a truncated version of sCR1 with greater inhibitory activity and an extended half-life (65). When compared to soluble human CR1 (TP10), CSL040 treatment prior to and after ischemic insult resulted in improved renal function, decreased incidence of tubular injury, and suppression of circulating C3b and C5a. Interestingly, biopsies from the CSL040 group showed decreased deposition of complement proteins and regulators from all pathways—C3d, C4d, C9, C1q, MBL, and Factor Bb—indicating robust complement suppression (66).

An alternative approach is to selectively inhibit upstream pathways. This approach is attractive as IRI in different organs seems to activate the complement system through distinct pathways. C1 INH blocks the classical and lectin pathways and is approved for clinical use in hereditary angioedema. In a brain-dead rat model, C1INH was found to reduce complement activation induced by brain death (67). In a murine renal IRI model, animals pre-treated with C1 INH have significantly better renal function and better survival. Less C5a release, C3b deposition and neutrophil/macrophage infiltration were observed. Interestingly, reduced tissue fibrosis and TGF-β1 levels were noted 30 and 90 days following the initial ischemic insult (68). In a nonhuman primate kidney transplant model, C1 INH has been shown to reduce complement deposition on biopsy and prevent DGF when delivered systemically to the brain-dead donor only (69) or recipient only (70). Lastly, in a porcine kidney autotransplant model, C1 INH treatment promoted more rapid glomerular function recovery and significantly prevented graft fibrosis in the long term (71).

#### 3.1.2 Other organs

Complement-targeting agents have also been studied in different organs to minimize IRI. Multiple groups have demonstrated the utility of C1 INH in liver IRI. Lehmann et al. showed, in a rat model, improved microvascular perfusion and decreased leukocyte-endothelial cell interaction on *in vivo* microscopy (72, 73). Heijnen et al. redemonstrated amelioration of hepatic IRI, reporting improved liver function and decreased circulating C4 with C1 INH treatment in a partial hepatic IRI rat model (74). Saidi et al. again used C1 INH in a partial IRI and 70% partial hepatectomy model, demonstrating a decrease in levels of IL-6, a key cytokine implicated in hepatic IRI pathogenesis. Interestingly, they also reported decreased IRI with C1 INH treatment of both wild type and C3 knockout mice, indicating that C1 INH does not act exclusively *via* blockade of classical activation (75). Clearly, upstream regulation of complement *via* C1 inhibition results in robust and reproducible amelioration of hepatic IRI.

sCR1 has also been utilized in other organs. In a rat liver transplant model, Chávez-Cartaya et al. reported improved hepatic function, decreased complement activity, and reduced C3 deposition in sCR1-treated rats (76). A similar study also demonstrated increased perfusion, decreased leukocyte adherence to endothelial cells, and decreased phagocytic activity of Kupffer cells, indicating an attenuation of several immunologic processes *via* complement modulation (77). In a rat lung transplant model, sCR1 improved oxygenation and isograft survival, and decreased circulating and deposited complement levels (78). Additionally, the sCR1-treated rats showed reduced neutrophil migration and lipid peroxidation (79).

Downstream proteins have also proved effective targets in the treatment of hepatic IRI. Kusakabe et al. targeted C5 with anti-C5 monoclonal antibody or C5aR1 antagonist in a murine model. Both approaches resulted in decreased platelet aggregation in hepatic microcirculation, expression of markers of apoptosis and necrosis, and ROS generation compared to controls (80). Another C5aR antagonist molecule trialed by Arumugam et al.—the same antagonist used in this group’s renal IRI study—ameliorated hepatic IRI in both a total and partial hepatic ischemia rat model (81). Both studies of C5aR blockade demonstrated reduction in inflammatory cytokine and chemokine secretion, as well as a decrease in the number of infiltrating immune cells such as macrophages and neutrophils. Zhang et al. took yet another approach to targeting C5 and its split products and succeeded using anti-C5a aptamers conjugated to an antioxidant to treat hepatic IRI in mice (82). As in the treatment of renal IRI, the targeting of C5, and particularly blockade of the C5a/C5aR interaction, decreases the severity of hepatic IRI.

While limited by the feasibility of clinical translation, cobra venom factor (CVF) is a C3 analogue that forms a stable C3/C5 convertase resistant to complement regulatory proteins and depletes components of the complement pathway effectively through unopposed complement activation *in vivo*. Rats pretreated with CVF had increased survival, decreased CH50 and MAC levels, circulating C5a, and reduced MAC deposition on biopsy (83). CVF has also been shown to reduce myocardial IRI, resulting in preservation of cardiac function and reduced complement deposition on histology (84).

The application of complement blockade for hepatic IRI requires consideration of the balance between tissue injury and regenerative potential, as complement plays an essential role in both processes. Marshall et al. explored this balance using a rat total IRI and 70% partial hepatectomy model. They found that inhibition of both MAC with CR2-CD59 and C3 with CR2-Crry ameliorate hepatic IRI; however, CR2-CD59 treatment leaves liver regeneration intact whereas CR2-Crry does not, indicating the importance of upstream complement activation in hepatic regeneration (85). While blockade of upstream targets such as C1 and C5 may successfully ameliorate hepatic IRI, the impact of these treatments on regenerative potential must also be taken into consideration. Taken together, available data show that IRI can be reduced by targeting various aspects of the complement pathways in a variety of organ systems.

#### 3.1.3 Clinical use

Several complement inhibitors have advanced to the clinic since the introduction of Eculizumab in 2007. Many have been tested off-label in the field of organ transplantation—mostly for antibody mediated rejection (AMR) (86–88). However, the importance of IRI is increasingly recognized as more marginal organs are utilized. In a phase I trial evaluating C1INH as an adjunct for highly sensitized kidney transplant patients following desensitization, Vo et al. noted lower rates of DGF among those that received C1 INH (10% vs 40%, n=10 in each arm) (86). The subsequent phase I/II RCT was performed in patients receiving high risk deceased-donor kidney allografts for the prevention of DGF. The treatment group received C1 INH intraoperatively and at 24 hours postoperatively. There was no significant difference in primary endpoint, defined as the need for hemodialysis within the first week following transplantation, patient or graft survival at one year. However, the treatment group required fewer dialysis sessions at 2 and 4 weeks posttransplant and had better renal function at 1-year compared to the placebo group (89). A three-year *post hoc* analysis of the trial revealed higher cumulative graft failure and gradually declining eGFR in the control group, with comparable patient survival compared to the C1 INH group (90).

In contrast, the results of Eculizumab trials for the reduction of IRI have been disappointing. Eculizumab has been extensively trialed for AMR prevention and treatment—including in sensitized recipients and HLA-incompatible transplants—with heterogenous results (91–95). A multi-center double-blinded RCT of peri-transplant eculizumab infusion in patients receiving extended criteria kidney allografts showed no difference in rate of DGF, short-term, or long-term graft function between the eculizumab and control groups (96). Additionally, the Alexion-sponsored PROTECT study (NCT02145182), a phase II/III multicenter RCT involving 288 patients from 77 centers that tested the efficacy of two doses of Eculizumab, one prior to and one after reperfusion, for DGF prevention, found no difference in DGF rate or graft survival (96). Interestingly, in a small single-center, non-blinded RCT (29 in the Eculizumab group and 28 in the control group) to assess eculizumab pretreatment in pediatric kidney transplantation, the authors demonstrated better eGFR on post-transplant day 1 and 2, and lower rates of arteriolar hyalinosis and chronic glomerulopathy on biopsy (97). However, several early graft losses were observed among eculizumab-treated children, including 4 within the first two months. Of note, 68% patients included in this study received living donor kidney transplant.

A few studies examined complement inhibitors in other solid organ transplant settings. TP-10, a sCR1, was evaluated in a multicenter double-blinded RTC among lung transplant recipients for the reduction of IRI. Although the incidence of operative deaths, infection and rejection, and length of hospital stay was not significantly different between the treatment and control groups, more patients that received pre-reperfusion TP-10 were extubated by 24-hour posttransplant, compared to controls (50% vs 19%). One study evaluated the use of Nafamostat, a synthetic broad spectrum protease inhibitor that acts on all three complement pathways, in liver transplantation and found a lower incidence of postreperfusion syndrome (98), although it is not clear whether this salutary effect was due to complement inhibition.

Taken together, these studies demonstrate the potential utility of complement blockade in the treatment of IRI, though the success of complement inhibitors in pre-clinical models has not directly translated to the clinical setting. Several issues have emerged. First, systemic administration to the recipient immediately prior to reperfusion may not allow sufficient time for the drug to take effect. Second, systemic administration poorly targets the end organ and may lead to undesired off-target effects. Third, complement activation precedes donor organ procurement and thus complement inhibition administered to recipient may not completely reverse complement activation that began in the donor. The question remains, at which level of the complement cascade should therapeutic agents target to best minimize IRI?

## 4 *Ex vivo* perfusion and drug delivery

The delivery of complement therapeutics for IRI can be achieved through multiple strategies. Donor preconditioning is commonly used in pre-clinical studies and is not without precedent in humans, as it generally takes a range of pharmacologic support to maintain a brain-dead donor (99); however, various ethical concerns have limited its application in clinical practice. Alternatively, complement therapeutics can be administered *ex vivo* or systemically during the peri-transplant period. *Ex vivo* delivery may limit off-target effects seen in systemic delivery.

*Ex vivo* machine perfusion has recently garnered interest as a method for marginal organ preservation and evaluation prior to transplantation. Several *ex vivo* machine perfusion devices are currently FDA approved for heart, lung, liver and kidney. The conditions used in *ex vivo* perfusion devices vary with respect to preservation temperature and perfusate composition, and are extensively reviewed elsewhere (100–102). One potential benefit of *ex vivo* machine perfusion is that therapeutics may be delivered to the target organ while on the circuit, a particularly attractive strategy for marginal organs. Major interventions, including stem cell therapy, anti-inflammatory and thrombolytic agents, and vasodilators in animal models were reviewed in great detail by Xu et al. (103). Using discarded human organs, several groups have evaluated therapeutic administration during machine perfusion, including high-dose antimicrobials to clear bacterial and fungal infections (104), ultraviolet light to reduce donor organ hepatitis C viral load (105, 106), Rituximab and novel chemokine-based immunotoxin to treat latent CMV-infected monocytes (107, 108), and thrombolytic therapy to preserve liver microvasculature (109).

As applied to complement therapeutics, *ex vivo* delivery *via* machine perfusion offers an opportunity to avoid potential deleterious effects of systemic complement blockade, while allowing for localized delivery of therapeutics that may have a greater potential to prevent or treat pathology compared to systemic blockade, especially in organs such as the kidney, lung, and liver that serve as sites of complement production. Local delivery and dosing can also be titrated to further avoid off-target effects and prolonged immunocompromise. Additionally, complement blockade in the donor or administered *ex vivo* also allows for termination of complement’s downstream effects at an earlier timepoint in the complement cascade. *Ex vivo* administration also provides an opportunity to monitor organ function and therapeutic efficacy simultaneously.

## 5 *Ex vivo* delivery of complement therapeutics for IRI

### 5.1 Pre-clinical models

Several studies to date have evaluated *ex vivo* delivery of complement therapeutics for IRI prevention in animal models. C5 is a commonly used target, as inhibition results in terminal blockade without compromising the utility of upstream proteins such as C3a, which is involved in opsonization. The administration of an anti-C5 mAb following prolonged cold ischemia time results in a decreased complement deposition in renal tissue and decreased creatinine in rat models (110, 111). Furthermore, addition of a localization sequence allows for complement blockade in the kidneys without decreasing circulating C5, highlighting the utility of tissue-specific localization to not compromise systemic complement function (111). *Ex vivo* administration of C5aR blockade has also successfully increased graft survival, as well as reduced tissue damage, apoptosis, and cytokine production (112). Antagonism of both C5 and the less central target C5aR improves graft function and decreases tissue injury.

Many have targeted C3 as it is involved in all three pathways and its split product C3a is highly inflammatory. In a murine renal transplant model, Zheng et al. administered an siRNA cocktail targeting C3, RelB, and Fas to the donor prior to graft procurement. Grafts then underwent static cold storage in the same siRNA cocktail. Renal function 48 hours post-transplant, as well as long-term survival was significantly better in the siRNA cocktail group compared to controls. Furthermore, siRNA solution reduced apoptosis of tubular cells and expression of inflammatory cytokines (113). siRNA remains an interesting approach to complement-targeting therapeutics that may be delivered *in vivo* or *ex vivo* with improvement in graft survival.

Mirococept (APT070) is a membrane-localizing complement inhibitor that consists of the minimal functional unit of human CR1 with a membrane binding tail designed to bind damaged cell membranes, which also blocks complement activation at the C3 level. Patel et al. perfused *ex vivo* rat kidney allografts with mirococept prior to subjecting the organs to prolonged cold ischemia time (114). This group first assessed tubular damage and complement deposition on histology and graft function with different lengths of cold ischemia time, finding increased C3 and C9 deposition and a dose-dependent rise in creatinine with increased cold ischemia time. Explanted rat donor kidneys were perfused with mirococept or control agent prior to cold storage and subsequent transplantation. This study reported better renal function at 24 and 72 hours in the treatment group, along with a 35% decrease in tubular damage and reduced complement deposition on histology (114). Indeed, C3 blockade results in robust suppression of inflammation and graft damage corresponding with improved graft function and prolonged survival. Given significant preclinical results, mirococept has since progressed to human trials, discussed below.

Complement-targeting therapeutics for IRI have been administered *ex vivo* in multiple other organ systems. Bergamachini et al. trialed C1 INH in a porcine liver reperfusion model. They demonstrated reduced complement activity, including generation of C3 and C3 deposition in the graft when C1 INH was added to the preservation solution following liver procurement. Biopsies of control organs had higher inflammatory cell infiltration, microabscess formation, and centrilobular necrosis compared to the treatment group, further supporting the potential of C1 INH to reduce inflammation in multiple organ systems and using multiple routes of administration (115).

Zheng et al. applied the aforementioned complement-silencing siRNA approach in a murine heart transplant model. siRNA solution containing TNF-α-, C3-, and Fas-silencing siRNA was administered to the donor prior to organ harvest. The organ was then preserved in and then flushed with the same siRNA cocktail, and then used for heterotopic transplantation. siRNA treatment resulted in successfully silenced C3 transcription. Additionally, 87.5% of siRNA-treated grafts preserved function at 100 days, compared to 0% in control groups (116). Wei et al. carried out a similar experiment in a porcine model, perfusing and storing cardiac allografts with siRNA targeting C3, caspases-8 and -3, and NF κB-p65, also finding improved graft survival and reduced tissue inflammation and apoptosis with treatment. Upon reperfusion, the cardiac allografts that received siRNA cocktail did not see a decline in hemodynamic parameters observed in controls (117). These experiments showcase the utility of *ex vivo* administration of complement—specifically C3—targeting siRNA for treatment of IRI in multiple organ systems.

Cheng et al. targeted C3aR in a murine DBD lung transplant model. C3aR is upregulated in lung parenchyma following brain death, thus presenting a particularly relevant target for lung IRI therapy. The authors administered a nebulized C3aR antagonist following brain death and prior to procurement, cold storage, and subsequent transplantation. Mice that received a C3aRA-treated graft had reduced IRI and acute rejection compared to untreated DBD. C3aRA treatment also decreased C3 and C3aR expression, injury on histology, and neutrophil and macrophage infiltration (118). Nebulizers offer a novel localized therapeutic delivery to the lung without the potentially deleterious effects of systemic treatment, that may also be delivered using an *ex vivo* machine perfusion circuit.

### 5.2 Clinical studies

To date, only one complement-targeting drug has been trialed using *ex vivo* delivery in humans. The EMPIRIKAL trial, a multi-center RCT, assessed the utility of mirococept when delivered *ex vivo via* machine perfusion, in prevention of IRI (119). 10mg mirococept was added to Soltran solution following initial flushing of the organ, and then administered *via* a single pass flush through the renal artery. The authors observed comparable levels of DGF between the mirococept and control groups, when stratified by site, pump, and donor type. Secondary outcomes, such as eGFR at one-year, acute rejection episodes, and duration of DGF were also comparable between the two groups (120). Of note, the authors failed to detect any mirococept or mirococept-specific IgG in the recipient serum. No CR1-specific staining above background levels of native CR1 was noted, and no significant difference in serum complement activity was observed between the treatment and control groups. The authors concluded that mirococept was likely not delivered at a clinically significant dose, and thus the study was terminated following the first cohort given the need for dose optimization. Kassimatis et al. conducted a follow-up dose-defining experiment using porcine kidneys, defining the optimal dose of mirococept (APT070) at 80 mg for *ex vivo* administration. Overall, the impact of complement inhibition in clinical kidney transplant when delivered *ex vivo* remains unclear; future studies using an optimized dose are needed to better evaluate the utility of this approach.

## 6 Conclusion

New solutions to the growing organ shortage, such as DCD and extended criteria organ transplants, have presented new challenges—namely increased vulnerability to IRI and increased rates of DGF, leading to worse outcomes compared to living and DBD organs. Complement activation is implicated in the pathogenesis of IRI across various organ systems and provides a promising target for IRI prevention. *Ex vivo* organ perfusion has emerged as a strategy not only for maintaining organs prior to transplantation, but also as a route of administration for pre-transplant therapies. In this review, we have highlighted the utility of complement blockade administered both systemically to the recipient and *ex vivo* to the target organ, with animal models exploring blockade at multiple levels in the complement pathway, including C1, C3, and C5. However, clinical studies so far have seen mixed results. C1INH administered to the recipient during the peri-transplant period may minimize IRI, especially among those receiving high KDPI kidneys. In contrast, human experience with eculizumab for IRI has been disappointing so far. The first human trial of *ex vivo* delivery of Mirococept also did not show a difference in DGF or graft survival, although mirococept drug dosing clearly requires optimization prior to continued evaluation. *Ex vivo* pre-treatment of complement blockade is a promising, yet imperfect strategy which requires further investigation prior to large-scale implementation in humans.

## Author contributions

QG and AB conceived this review. ID and QG preformed literature review and drafted the manuscript. IA, NA, and RK performed critical review and revision of the manuscript. MH and AB performed critical review and revision of the manuscript and oversaw the drafting process. All authors contributed to the article and approved the submitted version.

## Conflict of interest

The authors declare that the research was conducted in the absence of any commercial or financial relationships that could be construed as a potential conflict of interest.

## Publisher’s note

All claims expressed in this article are solely those of the authors and do not necessarily represent those of their affiliated organizations, or those of the publisher, the editors and the reviewers. Any product that may be evaluated in this article, or claim that may be made by its manufacturer, is not guaranteed or endorsed by the publisher.
